# Analytical Approximate Solutions for a General Class of Nonlinear Delay Differential Equations

**DOI:** 10.1155/2014/631416

**Published:** 2014-07-24

**Authors:** Bogdan Căruntu, Constantin Bota

**Affiliations:** Department of Mathematics, “Politehnica” University of Timişoara, Piaţa Victoriei 2, 300006 Timişoara, Romania

## Abstract

We use the polynomial least squares method (PLSM), which allows us to compute analytical approximate polynomial solutions for a very general class of strongly nonlinear delay differential equations. The method is tested by computing approximate solutions for several applications including the pantograph equations and a nonlinear time-delay model from biology. The accuracy of the method is illustrated by a comparison with approximate solutions previously computed using other methods.

## 1. Introduction

Delay differential equations are frequently used to model real-life phenomena in various fields such as mechanics, biology, computer science, and chemistry. Some of the recent studies involving delay differential equations include topics as varied as epidemic models that describe the fraction of a population infected by a virus [1], complex oscillator network [2], and neural networks [3].

It is known that the computation of exact solutions for delay differential equations is only possible in particular cases. It follows that, in most cases, in order to obtain information about the phenomena modeled, the computation of approximate solutions becomes a necessity.

In the present paper we used the polynomial least squares method (PLSM) to compute approximate solutions for the following class of nonlinear delay differential equations:

(1) F(x(n)(hn(t)),x(n−1)(hn−1(t)),x(n−2)(hn−2(t)),…,x(1)(h1(t)),x(h0(t)),t)=0,

together with the initial conditions

(2) $$\begin{array}{c} \overset{n-1}{\underset{i=0}{\sum }}r_{ij}x^{\left(i\right)}\left(\alpha \right)=s_{j},\;j\in 1,2,\ldots ,n. \end{array}$$

Here *F* is a function which satisfies such conditions as necessary to ensure that the problem (1)-(2) admits a unique solution, *r*_*ij*_ and *s*_*j*_ are real constants, and the functions *h*_*k*_(*t*), *k* ∈ 0,1,…, *n*, are polynomial functions in the *t* variable, *t* ∈ [*α*, *β*].

Among the methods recently used to compute approximate solutions for various delay differential equations of (1) type we mention the following.

- In [4] a numerical approximation based on the Bessel functions of the first kind was applied to (1) of the Riccati type.
- In [5] a two-stage order-one Runge-Kutta method was applied to (1) of the neutral-function differential type. The same equation was studied in [6, 7] by using the one-leg *θ*-method, in [8] by using the variational iteration method, in [9] by using the homotopy perturbation method, and in [10] by using a method based on Chebyshev polynomials.
- In [11] the homotopy perturbation method was applied to (1) of the pantograph type. The same equation was studied in [12] by using the variational iteration method.
- In [13] the Jacobi rational-Gauss collocation method was applied to (1) of the generalized pantograph type. The same equation was studied in [14] by using the Taylor series method, in [15] by using a Chebyshev method, and in [16] by using a Hermite collocation method.
- In [17] the variational iteration method and the Adomian decomposition method were applied for the case of a nonlinear time-delay model in biology which is also a particular case of (1).
- In [18, 19] local fractional methods (local fractional variation iteration method and local fractional Adomian decomposition method, resp.) were applied.

In the next section we introduce the polynomial least squares method (PLSM), which allows us to find analytical approximate polynomial solutions for the problem (1)-(2), and in the third section we compare our approximate solutions with approximate solutions presented in [4–17].

## 2. The Polynomial Least Squares Method

Let *D* be the operator associated with the differential equation (1):

(3) D(x)=F(x(n)(hn(t)),x(n−1)(hn−1(t)),x(n−2)(hn−2(t)),…,x(1)(h1(t)),x(h0(t)),t).

The error obtained by replacing the exact solution *x* with an approximate solution *x*_*α*_ is given by the so-called remainder:

(4) $$\begin{array}{c} R\left(t,x_{\alpha }\right)=D\left(x_{\alpha }\left(t\right)\right),\;t\in \left[\alpha ,\beta \right]. \end{array}$$

As a consequence, we will search for approximate polynomial solutions *x*_*α*_ of (1)-(2) on the [*α*, *β*] interval, solutions which satisfy the following conditions:

(5) $$\begin{array}{c} \left|R\left(t,x_{\alpha }\right)\right|<\epsilon ,\;\epsilon \in R_{+}, \end{array}$$

(6) $$\begin{array}{c} \overset{n-1}{\underset{i=0}{\sum }}r_{ij}\;x^{\left(i\right)}\left(\alpha \right)=s_{j},\;j\in 1,2,\ldots ,n. \end{array}$$

**Definition 1 .**

(a) We call* an ϵ-approximate polynomial solution* of the problem (1)-(2) an approximate polynomial solution *x*_*α*_ satisfying the relations (5) and (6).

(b) We call a* weak δ-approximate polynomial solution* of the problem (1)-(2) an approximate polynomial solution *x*_*α*_ satisfying the relation

(7) $$\begin{array}{c} \overset{\beta }{\underset{\alpha }{\int }}R^{2}\left(t,x_{\alpha }\right)dt\leq \delta ,\;\delta \in R_{+}, \end{array}$$

together with the initial condition (6).

**Remark 2 .**

Taking into account the way the problem (1)-(2) is defined, from the Weierstrass approximation theorem it follows that there exists the sequence of polynomials, *P*_*m*_(*t*) = *β*_0_ + *β*_1_ *t* + ⋯+*β*_*m*_ *t* ^*m*^, *β*_*i*_ ∈ *R*, *i* = 0,1,…, *m*, satisfying the conditions

(8) $$\begin{array}{c} \overset{n-1}{\underset{i=0}{\sum }}r_{ij}P_{m}^{\left(i\right)}\left(\alpha \right)=s_{j},\;j\in 1,2,\ldots ,n, \end{array}$$

such that the sequence of polynomials *P*_*m*_(*t*) is* convergent to the solution of the problem (1)-(2);* that is, lim⁡_*m*→*∞*_⁡*D*(*P*_*m*_(*t*)) = 0.

**Remark 3 .**

Any *ϵ*-approximate polynomial solution of the problem (1)-(2) is also a weak *ϵ* ^2^ · (*β* − *α*)-approximate polynomial solution, but the opposite is not always true. It follows that the set of weak approximate solutions of the problem (1)-(2) also contains the approximate solutions of the problem.

**Theorem 4 .**

The problem (1)-(2) admits a sequence of weak approximate polynomial solutions.

**Proof**

Taking into account the definition, we will find a weak *ϵ*-polynomial solution of the type

(9) $$\begin{array}{c} \tilde{x}\left(t\right)=\overset{m}{\underset{k=0}{\sum }}c_{k}\cdot t^{k},\;m>n, \end{array}$$

where the constants *c*_0_, *c*_1_,…, *c*_*m*_ are calculated using the following steps.

(1) In the first step we substitute the approximate solution (9) in (1) and obtain the remainder:
  (10) R(t,c0,c1,…,cm)=R(t,x~)=F(x~(n)(hn(t)),x~(n−1)(hn−1(t)),x~(n−2)×(hn−2(t)),…,x~(1)(h1(t)),x~(h0(t)),t).
(2) Next we compute *c*_0_, *c*_1_,…, *c*_*n*−1_ as functions of *c*_*n*_,…, *c*_*m*_ by using the initial conditions:
  (11) $$\begin{array}{c} \overset{n-1}{\underset{i=0}{\sum }}r_{ij}{\tilde{x}}^{\left(i\right)}\left(\alpha \right)=s_{j},\;j\in 1,2,\ldots ,n. \end{array}$$
(3) We attach to the problem (1)-(2) the following real functional:
  (12) $$\begin{array}{c} J\left(c_{n},\ldots ,c_{m}\right)=\overset{\beta }{\underset{\alpha }{\int }}R^{2}\left(t,c_{0},c_{1},\ldots ,c_{m}\right)dt. \end{array}$$
(4) Next we compute the values of *c*_*n*_^0^,…, *c*_*m*_^0^ as the values which give the minimum of the functional (12) and the values of *c*_0_^0^, *c*_1_^0^,…, *c*_*n*−1_^0^ again as functions of *c*_*n*_^0^,…, *c*_*m*_^0^ by using the initial conditions.
(5) By using the constants *c*_0_^0^, *c*_1_^0^,…, *c*_*m*_^0^ thus determined, we consider the polynomial:
  (13) $$\begin{array}{c} T_{m}\left(t\right)=\overset{m}{\underset{k=0}{\sum }}c_{k}^{0}\cdot t^{k}. \end{array}$$

Based on the way the coefficients of polynomial *T*_*m*_(*t*) are computed and taking into account the relations (10)–(13), the following inequality holds:

(14) $$\begin{array}{c} 0\leq \overset{\beta }{\underset{\alpha }{\int }}R^{2}\left(t,T_{m}\left(t\right)\right)dt\leq \overset{\beta }{\underset{\alpha }{\int }}R^{2}\left(t,P_{m}\left(t\right)\right)dt,\;\forall m\in N. \end{array}$$

It follows that

(15) $$\begin{array}{c} 0\leq \underset{m\to \infty }{\lim}\overset{\beta }{\underset{\alpha }{\int }}R^{2}\left(t,T_{m}\left(t\right)\right)dt\leq \underset{m\to \infty }{\lim}\overset{\beta }{\underset{\alpha }{\int }}R^{2}\left(t,P_{m}\left(t\right)\right)dt=0. \end{array}$$

We obtain

(16) $$\begin{array}{c} \underset{m\to \infty }{\lim}\overset{\beta }{\underset{\alpha }{\int }}R^{2}\left(t,T_{m}\left(t\right)\right)dt=0. \end{array}$$

From this limit we obtain that ∀*ϵ* > 0, ∃*m*_0_ ∈ *N* such that ∀*m* ∈ *N*, *m* > *m*_0_. It follows that *T*_*m*_(*t*) is a weak *ϵ*-approximate polynomial solution of the problem (1)-(2).

As a consequence of the second remark, in order to find *ϵ*-approximate polynomial solutions of the problem (1)-(2) by PLSM, we will first determine weak approximate polynomial solutions, ${\tilde{x}}_{\alpha }$ following the steps 1 to 5 previously described. If $\left|R(t,{\tilde{x}}_{\alpha })\right|<\epsilon$, then ${\tilde{x}}_{\alpha }$ is also an *ϵ*-approximate polynomial solution of the problem.

## 3. Applications

### 3.1. Application 1: Nonlinear Riccati Equation

Our first test problem is the following Cauchy problem:

(17) x′(t−2)−t2·x(2·t−3)−x2(t−1)+5t4−20t3 +19t2−2t−3=0,x(0)=−2.

The exact solution of this problem is *x*_*e*_(*t*) = *t* ^2^ − *t* − 2.

In [4], by using a numerical approximation based on the Bessel functions of the first kind, Yüzbaşi computed the following approximate solution of (17):

(18) $$\begin{array}{c} x_{\text{BES}}=\left(0.1\cdot 10^{-19}\right)t^{3}+t^{2}-1.0000000000000000001t-2. \end{array}$$

The maximum absolute error of this approximation is reported as 1.2171 · 10^−19^.

Using the steps described in the previous section we performed the following computations.

(i) We computed a polynomial solution of the form
  (19) $$\begin{array}{c} x_{\text{PLSM}}=c_{0}+c_{1}\cdot t+c_{2}\cdot t^{2}. \end{array}$$
(ii) Taking into account the fact that, by using the initial condition, *c*_0_ must be equal to −2, the functional (12) corresponding to the problem (17) is
  (20) J(c1,c2)=c145−2c13c23+6c135+6c12c227−134c12c221 +701c12105−c1c232+107c1c2214−1049c1c270+1963c1210 +c249−25c239+1206c2235−18341c2315+8543315.
(iii) To find the minimum of this functional we compute the stationary points as the solutions of the system
  (21) $$\begin{array}{c} \frac{\partial J}{\partial c_{1}}=0,\\ \frac{\partial J}{\partial c_{2}}=0. \end{array}$$
 Since the only stationary point is *c*_1_ = −1, *c*_2_ = 1 and it is easy to show that this point is indeed a minimum, we obtain the following polynomial approximate solution of (17):
  (22) $$\begin{array}{c} x_{\text{PLSM}}=-2-t+t^{2}, \end{array}$$
 which is actually the exact solution of the problem.

We remark that while in this simple case we were able to compute the exact minimum of the functional (12) in most of the applications the direct computation of the minimum is not possible and some numerical techniques are employed.

### 3.2. Application 2: Second-Order Neutral Functional-Differential Equation with Proportional Delays

Our second test problem is the following Cauchy problem:

(23) x′′(t)−34x(t)−x(t2)−x′(t2)−12x′′(t2)  +t2+t−1=0, 0≤t≤1,x(0)=0,  x′(0)=0.

The exact solution of this problem is *x*_*e*_(*t*) = *t* ^2^.

In [5], Bellen and Zennaro used a two-stage order-one Runge-Kutta method to compute a numerical solution of the problem (23) and the absolute error of their approximation is of the order 10^−3^. In [6, 7], Wang et al. used the one-leg *θ*-method to compute approximate solutions of (23) and the absolute error of their best approximation is of the order 10^−3^. In [8], Chen and Wang used the variational iteration method to compute approximate solutions of (23) and the absolute error of their best approximation is of the order 10^−6^. In [9], Biazar and Ghanbari used the homotopy perturbation method (HPM) to compute approximate solutions of (23) and the absolute error of their best approximation is of the order 10^−6^. In [10], Sedaghat et al. used a method based on Chebyshev polynomials to compute a numerical solution of the problem (23) and the absolute error of their approximation is of the order 10^−17^.

Using our method we performed the following computations.

(i) We compute a polynomial solution of the form
  (24) $$\begin{array}{c} x_{\text{PLSM}}=c_{0}+c_{1}\cdot t+c_{2}\cdot t^{2}. \end{array}$$
(ii) Taking into account the initial conditions we obtain the following values of the constants *c*_0_ = 0, *c*_1_ = 0. In this case the functional (12) corresponding to the problem (23) is
  (25) $$\begin{array}{c} J\left(c_{2}\right)=\frac{11c_{2}^{2}}{30}-\frac{11c_{2}}{15}+\frac{11}{30}. \end{array}$$
(iii) To find the minimum of this functional we compute the stationary points as the solutions of the equation *J*′(*c*_2_) = 0. The only stationary point is *c*_2_ = 1 and it is easy to show that this point is indeed a minimum.
(iv) We obtain the following polynomial approximate solution of (23):
  (26) $$\begin{array}{c} x_{\text{PLSM}}=t^{2}. \end{array}$$
 Again we obtained the exact solution of the problem.

### 3.3. Application 3: Pantograph-Type Nonlinear Equation

Our third test problem is the following Cauchy problem:

(27) x′′(t)−e−tx′(t−15)−(t−1)x(t)+2x(t3)−x2(t) −(−14sin2(t3)+49sin(t3)+sin(t9)−exp⁡(−t)×(16cos⁡(t3−115)−16sin(t2−110))−19cos⁡2(t2)+14cos⁡(t2)+23cos⁡(t6)−13sin(t3)cos⁡(t2)+t(−12sin(t3)−13cos⁡(t2)))=0,3x(0)+6x′(0)=2,−2x(0)+x′(0)=−12.

The exact solution of this problem is *x*_*e*_(*t*) = (1/2)sin(*t*/3) + (1/3)cos⁡(*t*/2).

In [11], Shakeri and Dehghan used the homotopy perturbation method (HPM) to compute approximate solutions *x*_HPM_ of (27).

In [12], Yildirim et al. used the variational iteration method (VIM) to compute approximate solutions *x*_VIM_ of (27).

Using our method we obtained the following polynomial approximate solution of (27):

(28) xPLSM=−3.8337691·10−6·t5+0.00088928·t4 −0.00309479·t3−0.0416659·t2+16·t+13.

Table 1 presents the comparison between the absolute errors (as the difference in absolute value between the approximate solution and the exact solution) corresponding to the approximate solution *x*_HPM_ from [11], to the approximate solution *x*_VIM_ from [12], and to our approximate solution *x*_PLSM_. The absolute errors corresponding to the approximate solution *x*_VIM_ are not explicitly given in [12], but we extracted some approximate values from a figure included in [12] (namely, Figure 2(b)).

It is easy to see that the approximate solution given by PLSM is much closer to the exact solution than the previous ones from [11, 12]. We mention the fact that our solution not only is more precise but also, at the same time, has a much simpler form.

### 3.4. Application 4: Generalized Pantograph-Type Equation

Our third test problem is the following Cauchy problem:

(29) $$\begin{array}{c} x^{\prime \prime \prime }\left(t\right)+x\left(t\right)+x\left(t-0.3\right)-e^{-t+0.3},\\ x\left(0\right)=1,\;x^{\prime }\left(0\right)=-1,\;x^{\prime \prime }\left(0\right)=1,\;0\leq t\leq 1. \end{array}$$

The exact solution of this problem is *x*_*e*_(*t*) = *e* ^−*t*^.

In [13], Doha et al. used the Jacobi rational-Gauss collocation method (JRC) to compute approximate solutions *x*_JRC_ of (29). In [14], Sezer and Akyuz-Dascioglu used the Taylor series method (TM) to compute approximate solutions *x*_TM_ of (29). In [15], Ozturk and Gulsu used a Chebyshev method (CM) to compute approximate solutions *x*_CM_ of (29). In [16], Yalçinbaş et al. used a Hermite collocation method (HCM) to compute approximate solutions *x*_HCM_ of the same equation.

Using our method we obtained the following polynomial approximate solution of (29):

(30) xPLSM=−1.6853299·10−6·t9+0.0000227752·t8 −0.000196271·t7+0.00138757·t6−0.00833288·t5 +0.0416666·t4−0.166667·t3+t22−t+1.

Table 2 presents the comparison between the absolute errors (as the difference in absolute value between the approximate solution and the exact solution) corresponding to the approximate solution *x*_TM_ from [14], to the approximate solution *x*_CM_ from [15], to the approximate solution *x*_HCM_ from [16], and to the (best) approximate solution *x*_JRC_ from [13], as given in [13], together with the errors corresponding to our approximate solution *x*_PLSM_.

Again it is easy to see that the approximate solution given by PLSM is much closer to the exact solution than the previous ones.

### 3.5. Application 5: Nonlinear Time-Delay Model in Biology

Our next test problem is

(31) $$\begin{array}{c} x^{\prime }\left(t\right)-2\cdot x\left(t\right)\left(1-\frac{x\left(t-0.1\right)}{0.5}\right)=0,\\ x\left(0\right)=1. \end{array}$$

The exact solution of this problem is not known.

In [17], Dehghan and Salehi used the variational iteration method (VIM) and the Adomian decomposition method (ADM) to compute approximate solutions *x*_VIM_ and *x*_ADM_ of (31).

Using our method we obtained the following polynomial approximate solution of (31):

(32) xPLSM=−2.57841·t7+15.8186·t6−39.5946·t5+52.059·t4 −38.6665·t3+16.3752·t2−3.89839·t+1.

Table 3 presents the comparison between the absolute errors (as the difference in absolute value between the approximate solution and the numerical solution presented in [17]) corresponding to the approximate solutions *x*_ADM_ and *x*_VIM_ from [17] and to our approximate solution *x*_PLSM_.

The approximate solution given by PLSM is closer to the numerical solution than the previous ones from [17].

### 3.6. Application 6: Scalar Differential Equation with Several Delays

Our last test problem is

(33) $$\begin{array}{c} x^{\prime }\left(t\right)+\frac{0.2}{\pi }x\left(t-\pi \right)\sin^{2}\left(t\right)+\frac{0.2}{\pi }x\left(t-2\pi \right)\cos^{2}\left(t\right)=0,\\ x\left(0\right)=1. \end{array}$$

The exact solution of this problem is not known. In [20], Berezansky and Braverman studied the existence of positive solutions for equations of the type

(34) $$\begin{array}{c} x^{\prime }\left(t\right)+\overset{m}{\underset{k=0}{\sum }}\alpha_{k}\left(t\right)x\left(h_{k}\left(t\right)\right)=0. \end{array}$$

In the case of (33) it was shown that there exists indeed such a positive solution, but the solution was not effectively computed.

Using our method on the interval [0,1] we obtained the following polynomial approximate solution of (33):

(35) xPLSM=−7.0502·10−6·t5−0.0000361911·t4+0.000175863·t3 +0.000388792·t2−0.00895158·t+0.1.

The error obtained by replacing the approximate solution back in the equation and computing the remainder is of the order 10^−7^.

It is easy to see that the solution is positive on the interval, where the computation was performed.

## 4. Conclusions

The polynomial least squares method (PLSM) was presented as a straightforward and efficient method to compute approximate polynomial solutions for nonlinear delay differential equations.

The applications presented clearly illustrate the accuracy of the method. Indeed, for the equations of the type (1) considered, namely, (17)–(33), the solutions obtained by using PLSM are more precise than the ones previously computed by using other methods. Moreover, for some problems PLSM was able to compute the exact solution while the other methods only produced approximate ones.

## Figures and Tables

**Table 1:** Comparison of VIM, HPM, and PLSM for (27).

*t*	VIM	HPM	PLSM
0.2	5 · 10^−5^	1.34 10^−5^	1.02 10^−8^
0.4	1 · 10^−4^	5.13 10^−5^	6.14 10^−8^
0.6	5 · 10^−5^	6.26 10^−6^	8.92 10^−8^
0.8	1 · 10^−4^	2.21 10^−5^	1.02 10^−7^
1	5 · 10^−4^	3.69 10^−5^	1.53 10^−7^

**Table 2:** Comparison of TM, CM, HCM, JRC, and PLSM for (29).

*t *	TM	CM	HCM	JRC	PLSM
0	0	0	0	0	0
0.2	8.54 · 10^−8^	3.70 10^−7^	6.200 10^−9^	3.605 10^−8^	6.255 10^−13^
0.4	5.36 · 10^−6^	2.38 10^−6^	5.760 10^−8^	9.299 10^−9^	7.193 10^−12^
0.6	5.95 · 10^−5^	5.97 10^−6^	1.796 10^−7^	3.503 10^−10^	1.839 10^−11^
0.8	3.26 · 10^−4^	3.48 10^−5^	3.735 10^−7^	8.345 10^−9^	3.341 10^−11^
1	1.21 · 10^−3^	2.03 10^−4^	6.368 10^−7^	1.161 10^−8^	5.642 10^−11^

**Table 3:** Comparison of VIM, HPM, and PLSM for (31).

*t*	ADM	VIM	PLSM
0	0	0	0
0.05	1.04 · 10^−1^	—	6.33 10^−2^
0.1	1.38 · 10^−1^	—	7.86 10^−2^
0.15	1.31 · 10^−1^	—	7.13 10^−2^
0.2	1.26 · 10^−1^	—	5.83 10^−2^
0.25	1.25 · 10^−1^	—	4.47 10^−2^
0.3	1.26 · 10^−1^	—	3.27 10^−2^
0.35	1.27 · 10^−1^	—	2.32 10^−2^
0.4	1.29 · 10^−1^	—	1.66 10^−2^
0.5	—	1.59 10^−2^	1.07 10^−2^
1	—	4.3 10^−3^	7.41 10^−4^
1.5	—	8.2 10^−4^	6.14 10^−4^
